# Diallyl Disulfide From Garlic Oil Inhibits *Pseudomonas aeruginosa* Quorum Sensing Systems and Corresponding Virulence Factors

**DOI:** 10.3389/fmicb.2018.03222

**Published:** 2019-01-07

**Authors:** Wen-Ru Li, Yong-Kai Ma, Xiao-Bao Xie, Qing-Shan Shi, Xia Wen, Ting-Li Sun, Hong Peng

**Affiliations:** State Key Laboratory of Applied Microbiology Southern China, Guangdong Provincial Key Laboratory of Microbial Culture Collection and Application, Guangdong Institute of Microbiology, Guangzhou, China

**Keywords:** diallyl disulfide, DADS, *Pseudomonas aeruginosa* PAO1, quorum sensing, virulence factors

## Abstract

Previously, we determined that diallyl disulfide (DADS) from garlic oil can inhibit *Pseudomonas aeruginosa* PAO1 pathogenic factors by inactivating the transcription of key genes from three quorum sensing (QS) systems (*las*, *rhl*, and *pqs*) based on the effects of DADS on growth, virulence factor production (elastase, pyocyanin, biofilm, and swarming motility), and RNA transcription (real-time q-PCR). To further investigate the mechanisms underlying the inhibition of the three *P. aeruginosa* QS systems by DADS, high-throughput RNA and proteome sequencing techniques were used to study differences in the transcriptional and proteome expression of *P. aeruginosa* PAO1 following treatment with DADS. The RNA-seq and proteomic data are available via NCBI Gene Expression Omnibus database with accession number GSE118801 and ProteomeXchange with identifier PXD011144, respectively. The experimental results indicated that all key genes of the three QS systems (*las*, *rhl*, and *pqs*) of *P. aeruginosa* PAO1 as well as the virulence factors (including exoprotease LasA, elastase LasB, lectin LecA and LecB, pyocyanin biosynthesis, and biofilm formation) regulated by these three QS systems were inhibited. This is consistent with our previous studies on the physiology, biochemistry, and RNA expression of *P. aeruginosa* treated with DADS. Additionally, our results also indicated that bacterial motility, chemotaxis, and two-component systems were inhibited by DADS treatment. All these changes abolish the sensitivity of *P. aeruginosa* PAO1 to environmental stimuli and cause the cells to be in a state of passivation. Further research is needed to determine how QS systems regulate these functions. Our findings could potentially contribute to the treatment and control of *P. aeruginosa* infection, virulence, and pathogenicity.

## Introduction

The extensive use of antibiotics has accelerated the formation of super resistant and multiple drug-resistant strains; thus, the strategies for prevention and control of pathogenic microbes must be changed (Woolhouse and Farrar, 2014). Quorum sensing (QS) has recently been established as an effective strategy for the treatment of pathogenic microbes. Many important pathogens regulate virulence via QS, which is a bacterial cell-to-cell communication system utilizing small signal molecules (Turan et al., 2017; Defoirdt, 2018; Prajapat and Saini, 2018). QS plays an important role in biofilm formation, pathogenicity, and virulence of pathogenic bacteria (Adonizio et al., 2006; Han et al., 2011; Husain et al., 2013; Hill and Liz-Marzán, 2017; Padder et al., 2018). The virulence and pathogenicity of pathogenic bacteria can be inhibited by inhibiting their QS. However, pathogenic growth is not inhibited and in this way QS inhibitors do not seem to provide favorable selection pressure for resistant bacteria. Previous studies have shown that many medicinal and edible plants produce QS inhibitors (Kalia, 2013; Kon and Rai, 2013).

*Pseudomonas aeruginosa* is a ubiquitous, clinically significant, Gram-negative bacillus and an opportunistic human pathogen (Stover et al., 2000; Lee et al., 2018). *P. aeruginosa* infections are particularly difficult to eradicate because this pathogen is prone to acquire resistance to multiple antibiotics and it prefers to form antibiotic-resistant biofilms (Galloway et al., 2012; He et al., 2018; Li et al., 2018). In recent years, a number of studies have reported that garlic extract contains *P. aeruginosa* QS inhibitors, including ajoene, a sulfur compound from garlic (Rasmussen et al., 2005; Bodini et al., 2009; Harjai et al., 2010; Jakobsen et al., 2012). In addition, garlic extract has been demonstrated to increase the fragility of *P. aeruginosa* biofilms and inhibit their formation (Bjarnsholt et al., 2005). Previously, we found that diallyl disulfide (DADS), an ingredient from garlic oil (Li et al., 2014), could decrease elastase and pyocyanin production, biofilm formation, and swarming motility in *P. aeruginosa* PAO1, but did not affect bacterial growth (Li et al., 2018). Real time q-PCR revealed that the related virulence genes were down-regulated by DADS. Moreover, DADS down-regulated several important genes (*lasI*, *lasR*, *rhlI*, *rhlR*, *pqsA*, and *pqsR*) of the *las*, *rhl*, and *pqs* QS systems. Therefore, we concluded that DADS inhibits *P. aeruginosa* PAO1 pathogenic factors by inactivating the key genes of *las*, *rhl*, and *pqs* QS systems (Li et al., 2018).

In this study, to further investigate the mode of action of DADS in *P. aeruginosa* PAO1, we analyzed differences in transcriptome and proteome expression between untreated and DADS-treated *P. aeruginosa* PAO1 using high-throughput sequencing technology. Our research results may have important potential implications for the treatment of *P. aeruginosa* infection and for controlling its virulence and pathogenicity.

## Materials and Methods

### Chemical Reagents, Microorganisms, Media, and Cultivation

Strain *P. aeruginosa* PAO1, the DADS reagent, and Luria-Bertani (LB) medium were the same as in our previous study (Li et al., 2018). All solvents and reagents were of analytical grade unless otherwise specified.

### Effects of DADS on *P. aeruginosa* PAO1 Transcriptome Based on RNA High-Throughput Sequencing

Luria-Bertani medium (50 mL) was inoculated with exponential growth phase *P. aeruginosa* PAO1 at a concentration of 10^8^ CFU/mL. DADS was then added at a concentration of either 0 (control) or 0.64 mg/mL, in triplicate. All six experiment groups were incubated in a water bath shaker at 37°C with a shaking rate of 180 rpm for 5 h. Cells were then sampled and centrifuged from the three control groups and three DADS treatment groups. The cell precipitates in the control and DADS-treated groups were separately snap-frozen at -80°C.

#### RNA Sample Preparation

Total RNA was isolated from cells using TRIzol (ThermoFisher Scientific, Inc., United States) according to the manufacturer’s protocol. RNA purity was determined using a NanoPhotometer spectrophotometer (IMPLEN, CA, United States). All RNA samples had an A260:A280 ratio between 1.8 and 2.0. RNA concentration was measured using a Qubit RNA Assay Kit with a Qubit 2.0 Fluorometer (Life Technologies, CA, United States). RNA integrity was evaluated using the RNA Nano 6000 Assay Kit of the Agilent Bioanalyzer 2100 system (Agilent Technologies, CA, United States); all samples had RNA integrity > 7.0.

#### Library Preparation for Strand-Specific Transcriptome Sequencing

RNA sequencing was accomplished by Beijing Novogene Bioinformatics Technology Co., Ltd. (Beijing, China). A total of 3 μg RNA per sample was used as input material for RNA sample preparation. Sequencing libraries were generated using the NEBNext Ultra Directional RNA Library Prep Kit for Illumina (NEB, United States) following the manufacturer’s recommendations and index codes were added to attribute sequences to each sample. Briefly, mRNA was purified from total RNA using poly-T oligo-attached magnetic beads. The rRNA was removed using a specialized kit that leaves the mRNA. Fragmentation was carried out using divalent cations under elevated temperature in NEBNext First Strand Synthesis Reaction Buffer (5×). First strand cDNA was synthesized using a random hexamer primer and M-MuLV Reverse Transcriptase (RNaseH). Second strand cDNA synthesis was subsequently performed using DNA polymerase I and RNase H. PCR was then performed with Phusion High-fidelity DNA polymerase, universal PCR primers, and Index primers. Finally, products were purified (AMPure XP system) and library quality was assessed on an Agilent Bioanalyzer 2100 system. RNA sequencing was performed at Novogene Co., Ltd. (Beijing, China) on an Illumina Hiseq platform.

#### Reads Mapping to the Reference Genome, Novel Gene and Gene Structure Analysis, and Quantification of Gene Expression Levels

The reference genome and gene model annotation files were directly downloaded from the genome website of NCBI^1^. Both reference genome index building and aligning of the clean reads to the reference genome were performed using bowtie2-2.2.3 (Langmead and Salzberg, 2012). Rockhopper was used to identify novel genes, operons, and transcription start sites. This system can be used for efficient and accurate analysis of bacterial RNA-seq data, thus assisting with the interpretation of bacterial transcriptomes (McClure et al., 2013). Next, the sequences 700 bp upstream of the transcription start sites were extracted for promoter prediction using Time-Delay Neural Network (TDNN). HTSeq v0.6.1 was used to count the number of reads that mapped to each gene, and the Fragments Per Kilobase of exon model per Million mapped reads (FPKM) for each gene was then calculated based on the length of the gene and the reads counts that mapped to the gene (Langmead et al., 2009).

#### Differential Expression Analysis and GO and KEGG Enrichment Analysis of Differentially Expressed Genes

Differential expression analysis of the two groups was performed using the DESeq R package (1.18.0). The resulting *P*-values were adjusted using Benjamini and Hochberg’s method for controlling the false discovery rate. Genes with an adjusted *P*-value < 0.05 identified by DESeq were assigned as differentially expressed. Gene Ontology (GO) enrichment analysis of differentially expressed genes was implemented using the GOSeq R package, which corrects for gene length bias. GO terms with corrected *P*-value < 0.05 were considered significantly enriched by differentially expressed genes. The Kyoto Encyclopedia of Genes and Genomes (KEGG) database is a resource for deciphering the functions and abilities of various biological systems based on molecular-level information, especially large-scale molecular datasets generated by high-throughput experimental technologies including genome sequencing^2^. KOBAS software was used to test the statistical enrichment of differentially expressed genes in KEGG pathways (Mao et al., 2005).

#### Accession Number

The RNA-seq datasets are available at the NCBI Gene Expression Omnibus (GEO) database under accession number GSE118801.

### Effects of DADS on the Proteome of *P. aeruginosa* PAO1 by iTRAQ High-Throughput Sequencing Analysis

To establish the effect of DADS on the *P. aeruginosa* proteome, 50-mL LB medium samples were inoculated at 10^8^ CFU/mL with exponential growth phase *P. aeruginosa* PAO1. DADS was then added at a concentration of 0 (control) or 0.64 mg/mL, in triplicate. The six experimental groups were incubated for 5 h in a water bath shaker at 37°C with a shaking rate of 180 rpm. Cells from the three control groups and three DADS treatment groups were then sampled and centrifuged. The cell precipitate from each experiment group was snap-frozen at -80°C.

#### Sample Preparation and iTRAQ Labeling

Protein extracts were cleaned up using the RIPA reagent. Protein concentration was estimated by Bradford assay using BSA as the standard (BCA Assay Kit, Biotech) according to the manufacture’s protocol. Protein iTRAQ sequencing analysis was performed at Guangzhou FitGene Biotechnology Co., Ltd. (Guangzhou, China). Proteins were digested according to the FASP method (Wisniewski et al., 2009; Lan et al., 2012; Kambiranda et al., 2014). iTRAQ labeling was performed using the iTRAQ reagents – 8plex Kit (Applied Biosystems, SCIEX) according to the user’s manual. The three control *P. aeruginosa* PAO1 samples were labeled with iTRAQ tags 114, 115, and 116, while the three *P. aeruginosa* PAO1 samples treated with DADS were labeled with iTRAQ tags 117, 118, and 119.

#### High-pH Reversed-Phase Chromatography

iTRAQ labeled samples were diluted to 100 μL with 20 mM HCOONH_4_ and 2 M NaOH (pH 10) prior to HPLC. Peptides were separated by a linear gradient consisting of 20 mM HCOONH_4_, 2 M NaOH, and 80% CAN, from 5 to 40% of mobile phase B for 30 min at a flow rate of 0.2 mL/min. The UV detector was set at 214/280 nm, and fractions were collected every 1 min. In total, 10 fractions were pooled for each sample and dried using a vacuum centrifuge.

#### RPLC-MS Analysis

Peptides were acidified with 50% CF_3_COOH, and then separated by a linear gradient from 5 to 40% of mobile phase B for 70 min at a flow rate of 300 mL/min. MS analysis was performed on a TripleTOF 5600 system (AB SCIEX) in information dependent mode. MS spectra were acquired across the mass range of 400–1250 m/z in high resolution mode using 250 ms accumulation time per spectrum. A maximum of 50 precursors per cycle was chosen for fragmentation from each MS spectrum with 100 ms minimum accumulation time for each precursor and dynamic exclusion for 20 s. Tandem mass spectra were recorded in high sensitivity mode with rolling collision energy on and iTRAQ reagent collision energy adjustment on.

#### Data Analysis

Relative quantification and protein identification were performed with ProteinPilot software (version 4.0, revision 148085, Applied Biosystems) using the Paragon algorithm as the search engine. Specified processing included quantitate, bias correction, and background correction. All proteins identified had ≥95% confidence, and the protein confidence threshold cutoff was set to 1.3 (unused) with at least more than one peptide above the 95% confidence level. To designate significant changes in protein expression, fold-changes > 2 or < 0.5 were set as cutoff values. The differentially expressed proteins were examined using QuickGO (^3^Annotation Blacklist^4^) for GO annotation and enrichment analyses. The GO project classifies proteins in three categories based on their annotation: biological process, cellular component, and molecular function. Following annotation, the enzyme codes assigned to each protein were sequentially mapped to the known metabolic pathways in KEGG^2^ for biological pathway analysis.

#### Accession Number

The mass spectrometry proteomics data have been deposited to the ProteomeXchange Consortium via the PRIDE partner repository with the dataset identifier PXD011144.

## Results

### The Effects of DADS on *P. aeruginosa* PAO1 Transcription

**FIGURE 1:**
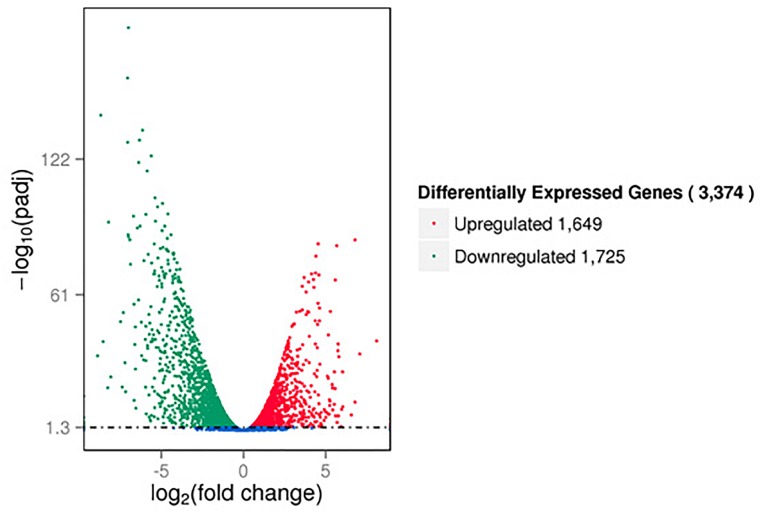
Volcano plots of differentially expressed genes based on RNA sequencing analysis of untreated and DADS-treated *Pseudomonas aeruginosa* PAO1. Each gene is represented by a dot in the graph. The *x*-axis and *y*-axis represent the log_2_ value of the fold change and the t-statistic as –log_10_ of the *p*-value, respectively. The genes represented in red (upregulated) and green (downregulated) are differentially expressed genes with >twofold change and a *p*-value < 0.05.

The RNA-seq results showed that a large number of *P. aeruginosa* PAO1 genes were differentially expressed following DADS treatment. We found that more than 50% of all detected genes (3374 of 5630 genes) were more than twofold differentially expressed following DADS treatment (Supplementary Tables S1–S3). The volcano plots of differentially expressed genes (Figure 1) showed that the differentially expressed genes were either more than twofold downregulated (green dots, 1,725 genes) (Supplementary Table S4) or upregulated (red dots, 1,649 genes) (Supplementary Table S5).

**FIGURE 2:**
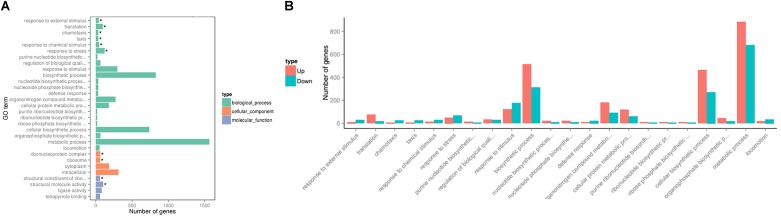
Significantly enriched Gene Ontology categories (*q*-value < 0.05) of differentially expressed genes based on RNA sequencing analysis of the DADS-treated group compared to the control group. Three main categories were identified: biological process, cellular component, and molecular function. **(A)** the most highly enriched GO terms; **(B)** the most highly enriched GO terms in the biological process category.

To investigate changes in *P. aeruginosa* PAO1 gene expression patterns following DADS treatment, the percentage of genes in each GO category was analyzed. The GO categories significantly enriched (q < 0.05) among the differentially expressed genes are shown in Figure 2. Within the biological process category, 22 terms were enriched in the differentially expressed genes, while only four terms were enriched in the cellular component category and the molecular function category, respectively (Figure 2A). In the biological process category, “response to external stimulus,” “translation,” “chemotaxis,” “taxis,” “response to chemical stimulus,” and “response to stress” were the most significantly enriched terms. The terms “response to stimulus,” “chemotaxis,” “taxis,” “response to chemical stimulus,” “response to stress,” “response to external stimulus,” and “locomotion” contained more downregulated than upregulated genes, whereas upregulated genes were more prevalent than downregulated genes in the other significantly enriched terms (Figure 2B).

**FIGURE 3:**
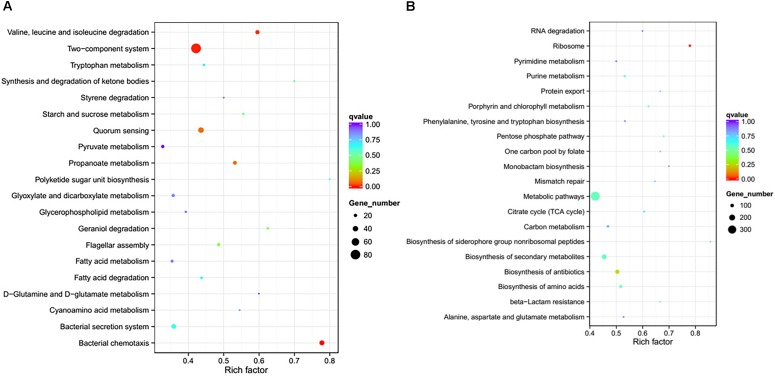
Significantly enriched KEGG pathway scatter plot (0.00 < *q*-value < 1.00) of differentially expressed genes based on RNA sequencing analysis of the DADS-treated group compared to the control group. **(A)** downregulated genes; **(B)** upregulated genes.

To further investigate the biological functions of the differentially expressed genes, KEGG analysis was performed to classify the functions of the identified genes (Figure 3). The downregulated genes were significantly enriched in the following KEGG pathway terms (Figure 3A): Bacterial chemotaxis, Two-component system, Valine, leucine and isoleucine degradation, Propanoate metabolism, QS, Flagellar assembly, Geraniol degradation, Synthesis and degradation of ketone bodies, Starch and sucrose metabolism, and Bacterial secretion system. The upregulated genes were significantly enriched in the following KEGG pathway terms (Figure 3B): Ribosome, Biosynthesis of antibiotics, Biosynthesis of amino acids, Porphyrin and chlorophyll metabolism, Biosynthesis of secondary metabolites, beta-Lactam resistance, Pentose phosphate pathway, Metabolic pathways, Purine metabolism, and Citrate cycle (TCA cycle).

### The Effects of DADS on *P. aeruginosa* PAO1 Proteome

**FIGURE 4:**
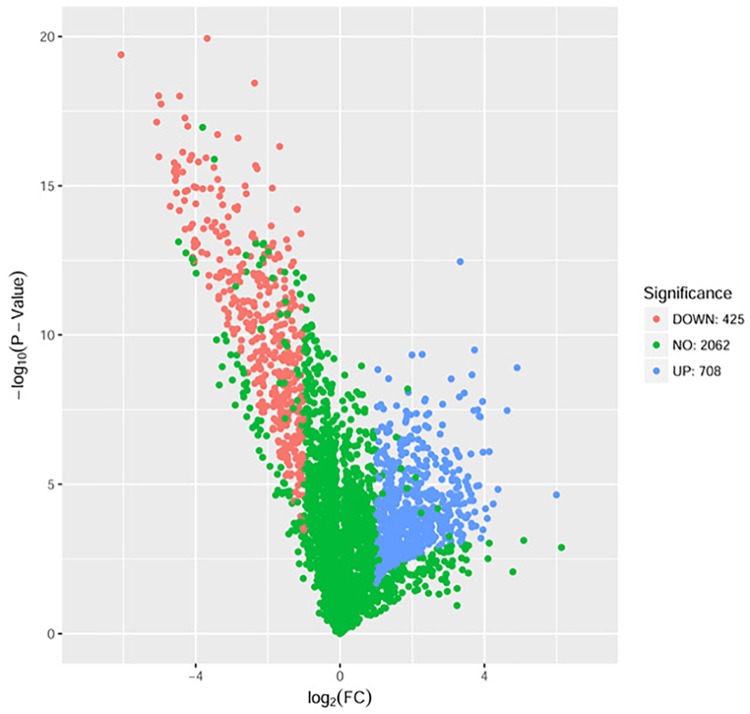
Volcano plots of the differentially expressed genes based on proteome sequencing analysis of untreated and DADS-treated *P. aeruginosa* PAO1. Each protein is represented by a dot in the graph. The *x*-axis and *y*-axis represent the log_2_ value of fold change and the t-statistic as –log_10_ of *p*-value, respectively. The genes represented in blue (up regulated) and red (down regulated) are differentially expressed genes with >twofold change and a *p*-value < 0.05.

**FIGURE 5:**
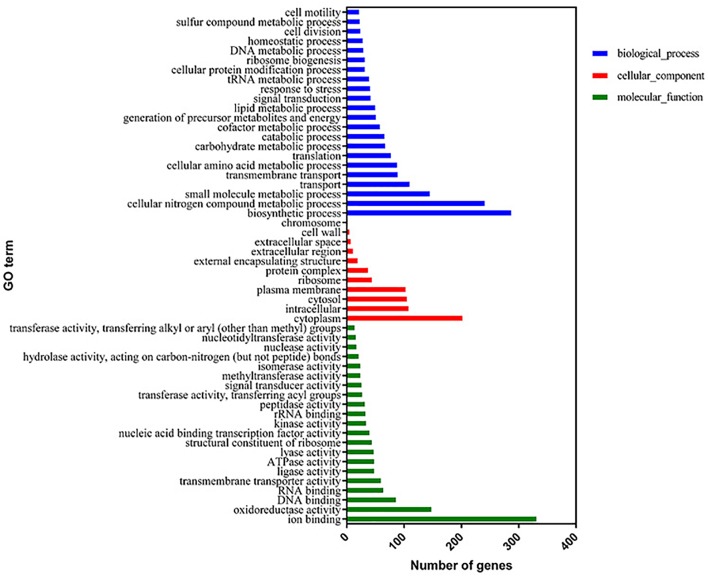
Significantly enriched Gene Ontology categories (*q*-value < 0.05) of differentially expressed genes based on proteome sequencing analysis of the DADS-treated group compared to the control group. Three main categories were identified: biological process, cellular component, and molecular function.

The proteome sequencing results revealed that more than 30% of all detected proteins (1133 of 3304 proteins) were more than twofold differentially expressed following DADS treatment (Supplementary Tables S6, S7). The volcano plots of differentially expressed proteins (Figure 4) showed that 425 proteins were down-regulated (red dots) and 708 proteins were up-regulated (blue dots) > twofold following DADS treatment (Supplementary Tables S6, S7). The GO categories significantly enriched (q < 0.05) among those differentially expressed genes are shown in Figure 5. Within the biological process category, 22 terms were enriched in differentially expressed genes, while 11 and 21 terms were enriched in the cellular component category and the molecular function category, respectively. Most of the terms in the biological process category were related to metabolism. Of these, the terms “response to stress,” “cell motility” and “signal transduction” were consistent with the GO analysis of the RNA high-throughput sequencing. The term “sulfur compound metabolic process” is significant for understanding the mechanism of DADS in *P. aeruginosa* PAO1.

**FIGURE 6:**
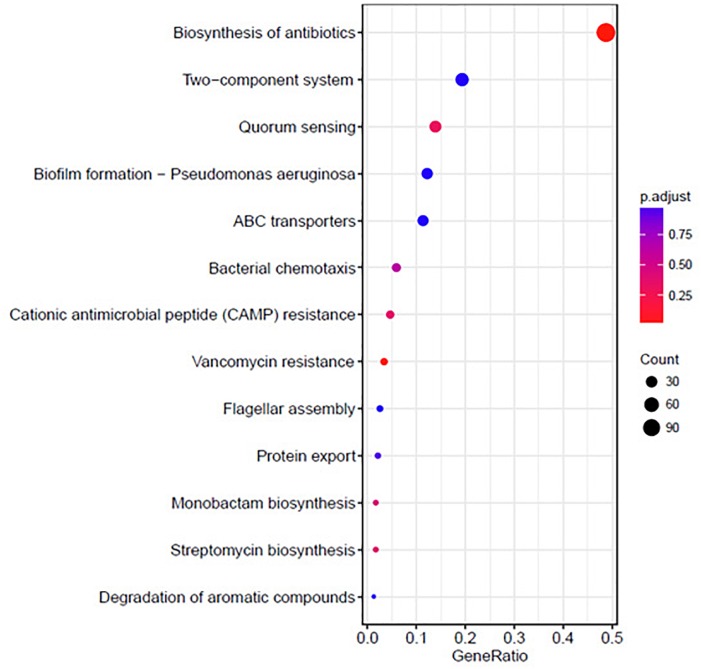
Significantly enriched KEGG pathway scatter plot (0.00 < *q*-value < 1.00) of differentially expressed genes based on proteome sequencing analysis of the DADS-treated group compared to the control group.

The KEGG analysis results are shown in Figure 6. The differentially expressed genes were significantly enriched in the following KEGG pathway terms: Biosynthesis of antibiotics, Two-component system, QS, Biofilm formation, ABC transporters, Bacterial chemotaxis, Cationic antimicrobial peptide (CAMP) resistance, Vancomycin resistance, Flagellar assembly, and Protein export.

## Discussion

**FIGURE 7:**
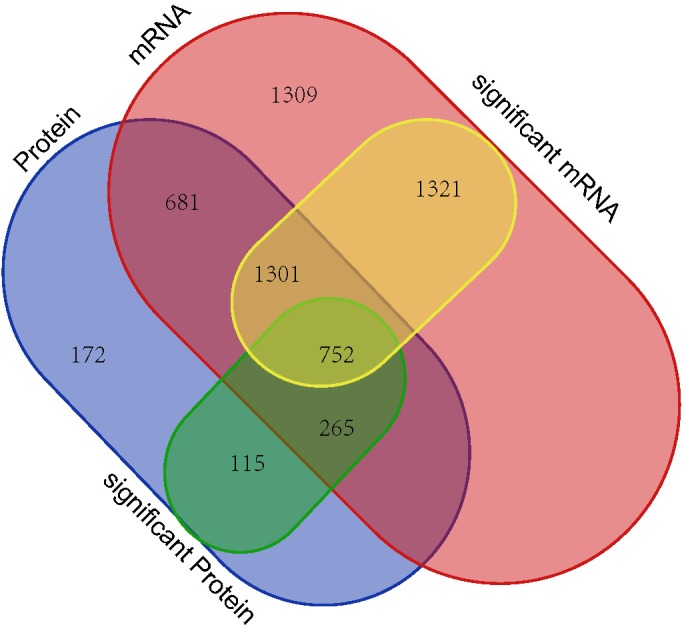
Venn diagram showing all identified, as well as all significantly enriched, mRNAs and proteins and their overlaps.

The RNA and proteome high-throughput sequencing results showed that > 3,000 genes (1,725 down-regulated and 1,649 up-regulated genes) and 1133 proteins (425 down-regulated and 708 up-regulated proteins) were differentially expressed in *P. aeruginosa* PAO1 following DADS treatment, respectively. These results indicate that DADS treatment has an immense influence on *P. aeruginosa* PAO1 gene expression. Comparison of the iTRAQ and RNA-seq data revealed that the number of differentially expressed genes of the RNA-seq data was 2.97 times higher than that of the iTRAQ data (Supplementary Tables S2, S7). This suggested that RNA-seq is more sensitive at detecting gene expression than iTRAQ. RNA sequencing can identify more expressed genes than proteomic analysis. When integrating these datasets, we identified 752 genes with significant differences following DADS treatment at both the mRNA and protein levels as shown in Venn diagram (Figure 7). The Venn diagram in Figure 7 shows the differentially expressed genes are highly consistent in RNA-seq and proteomic analysis, though it has been widely reported that the correspondence of transcriptomic and proteomic data is low due to the numerous and complex regulatory mechanism in gene transcription and protein synthesis (Dressaire et al., 2010; Haider and Pal, 2013; Margalef-Català et al., 2016).

**FIGURE 8:**
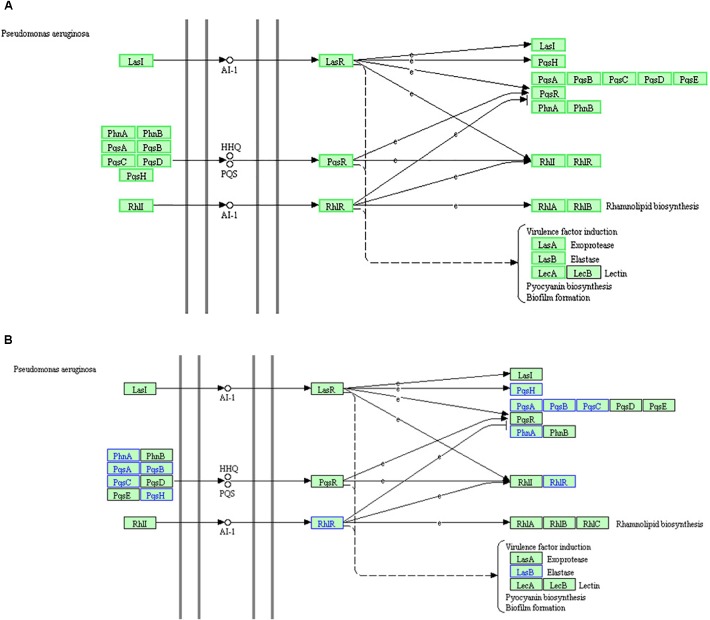
The KEGG “quorum sensing” pathway based on RNA high-throughput sequencing analysis **(A)** and proteome high-throughput sequencing analysis **(B)**. **(A)** The green box proteins indicate that the RNA expression of the gene is down regulated, while the black box proteins show no changes in RNA expression. **(B)** Proteins in blue indicate downregulated expression, while proteins in black indicate no change in expression.

Kyoto Encyclopedia of Genes and Genomes and GO analyses of the proteome and RNA high-throughput sequencing results were consistent. Based on these data, the “QS” pathway in *P. aeruginosa* is the most significant KEGG pathway among the enriched downregulated genes. However, the KEGG “QS” pathway, is not enriched in the corresponding GO term because this term is not listed in the GO database. The KEGG “QS” pathway in RNA high-throughput sequencing analysis (A) and proteome high-throughput sequencing analysis (B) is shown in Figure 8. There are three different QS systems in *P. aeruginosa*, *las*, *rhl*, and *pqs* (Whiteley et al., 1999; Waters and Bassler, 2005; Wilder et al., 2011; Guo et al., 2014), which are hierarchically arranged; the *las* system positively regulates both the *rhl* and *pqs* systems, and the *rhl* and *pqs* systems regulate each other (Figure 8). In the *las* system (Passador et al., 1993; Finch et al., 1998), LasI catalyzes the synthesis of the signal molecule (AI-1), which binds LasR and activates the expression of several target genes, including *lasI* (suggesting that the *las* system positively regulates itself); *pqsH*, *pqsA*, *pqsB*, *pqsC*, *pqsD*, *pqsE*, *pqsR*, *phnA*, and *phnB* (suggesting that the *las* system positively regulates the *pqs* system); and *rhlI* and *rhlR* (suggesting that the *las* system positively regulates the *rhl* system). In the *pqs* system (Williams and Cámara, 2009), PhnA, PhnB, PqsA, PqsB, PqsC, PqsD, and PqsH are combined to catalyze the synthesis of the signal molecules (HHQ or PQS), which bind PqsR and activate the expression of several genes, including *pqsR* (suggesting that the *pqs* system positively regulates itself), *rhlI*, and *rhlR* (suggesting that the *pqs* system positively regulates the *rhl* system). In the *rhl* system (Pearson et al., 1995; Latifi et al., 1996), RhlI catalyzes the synthesis of the signal molecule (AI-1), which binds RhlR and activates the expression of several genes, including *pqsR*, *phnA*, and *phnB* (suggesting that the *rhl* system positively regulates the *pqs* system); *rhlI* and *rhlR* (suggesting that the *rhl* system positively regulates itself); and *rhlA* and *rhlB* (suggesting that the *rhl* system positively regulates rhamnolipid biosynthesis). Furthermore, Figure 8A also shows that the virulence factors LasA (exoprotease) and LasB (elastase), LecA and LecB (lectin), pyocyanin biosynthesis, and biofilm formation are co regulated by the *las*, *pqs*, and *rhl* systems.

RNA analysis of the “QS” pathway (Figure 8A) demonstrated that the key genes of the three QS systems were downregulated. Elastase production, pyocyanin biosynthesis, and biofilm formation were also downregulated. These results are consistent with the q-PCR results and physiological observations in our previous study (Li et al., 2018). DADS inhibits the expression of all key genes of the three hierarchical QS systems, and thus inhibits the production of virulence factors regulated by the QS systems.

Both the RNA and proteome high-throughput sequencing GO and KEGG analysis results showed that the downregulated genes were also significantly enriched in bacterial chemotaxis, bacteria motility, and two-component systems. GO analysis demonstrated that the response to stimulus (response to stimulus, response to stress, response to chemical stimulus, and response to external stimulus terms), chemotaxis (chemotaxis and taxis terms), and bacterial motility (locomotion term) related genes were most highly downregulated. Furthermore, KEGG analysis also showed that the downregulated genes were significantly enriched in the KEGG pathway terms “Bacterial chemotaxis,” “Flagellar assembly,” and “Two-component system.”

The downregulated genes were enriched in “bacterial chemotaxis” both in the GO terms and KEGG pathways. Bacterial chemotaxis is the movement of bacterial cells in response to chemical stimuli (Hazelbauer, 2012; Parkinson, 2013). For example, bacterial cells direct their movements according to certain chemicals in their environment. This is important for bacteria to find food or to flee from toxins by swimming motility toward or away from certain chemical molecules. Therefore, bacterial chemotaxis depends on bacterial motility and they are closely related. The KEGG pathway term “Flagellar assembly” is closely related to GO term “bacterial motility.” The downregulated genes were enriched in the “flagellar assembly” KEGG pathway and “bacterial motility” GO term. This indicates that bacterial motility was inhibited following DADS treatment. These results were consistent with our previous study (Li et al., 2018) demonstrating that DADS inhibited the swarming motility of *P. aeruginosa* PAO1 based on physiological observations. The “two-component system” KEGG pathway is also known as “two-component signal transduction system,” which enables bacteria to sense, respond, and adapt to environmental or intracellular changes (Stock et al., 2000; Mascher et al., 2006; Capra and Laub, 2012). Two-component systems are responsible for environmental information processing and signal transduction. Therefore, the enriched GO term “response to stimulus” was consistent with the “two-component system” KEGG pathway. Following DADS treatment, *P. aeruginosa* PAO1 was no longer sensitive to environmental changes and stimulation. The enrichment of downregulated genes in the terms “bacterial chemotaxis,” “two-component system,” and “bacterial motility,” suggests that *P. aeruginosa* PAO1 cells were in a “passivation” state following DADS treatment.

Additionally, the enriched GO term “sulfur compound metabolic process” in the proteome high-throughput sequencing analysis was significant for understanding the mechanism of action of DADS in *P. aeruginosa* PAO1. These experimental results provide directions for further studies.

In summary, based on RNA and proteome high-throughput sequencing analysis, following DADS treatment, all key genes of the three QS systems (*las*, *rhl*, and *pqs*) of *P. aeruginosa* PAO1 were inhibited and thus the virulence factors (including exoprotease LasA, elastase LasB, pyocyanin biosynthesis, and biofilm formation) regulated by these three QS systems were also inhibited. The QS systems of *P. aeruginosa* PAO1 constituted the most significant pathway inhibited by DADS treatment. Additionally, bacterial motility, chemotaxis, and two-component systems were also inhibited by DADS treatment. All these changes reduce the sensitivity of *P. aeruginosa* PAO1 to environmental stimuli and cause the cells to enter a passivation state. Further research is needed to determine whether and how bacterial motility, chemotaxis, and two component systems are regulated by the QS systems.

## Author Contributions

W-RL conceived and supervised the study and wrote the paper. W-RL, Y-KM, and XW performed all the experiments. W-RL and Y-KM carried out the data analyses. X-BX, Q-SS, T-LS, and HP contributed reagents or materials.

## Conflict of Interest Statement

The authors declare that the research was conducted in the absence of any commercial or financial relationships that could be construed as a potential conflict of interest.

## Footnotes

1 https://www.ncbi.nlm.nih.gov/nuccore/AE004091.2

2 http://www.genome.jp/kegg/

3 http://www.ebi.ac.uk/QuickGO/Dataset.html

4 http://www.ebi.ac.uk/QuickGO/AnnotationBlacklist.html

## Supplementary Material

TABLE S1 Gene description of DADS-treated *Pseudomonas aeruginosa* PAO1 vs. control groups based on RNA-seq analysis.

TABLE S2 Differentially expressed gene data of DADS-treated *Pseudomonas aeruginosa* PAO1 vs. control groups based on RNA-seq analysis.

TABLE S3 Differential analysis results of DADS-treated *Pseudomonas aeruginosa* PAO1 vs. control groups based on RNA-seq analysis.

TABLE S4 The downregulated gene data of DADS-treated *Pseudomonas aeruginosa* PAO1 vs. control groups based on RNA-seq analysis.

TABLE S5 The upregulated gene data of DADS-treated *Pseudomonas aeruginosa* PAO1 vs. control groups based on RNA-seq analysis.

TABLE S6 Protein data of DADS-treated *Pseudomonas aeruginosa* PAO1 vs. control groups based on proteome sequencing analysis.

TABLE S7 The twofold differentially expressed protein data of DADS-treated *Pseudomonas aeruginosa* PAO1 vs. control groups based on proteome sequencing analysis.
